# Efficacy and safety of ethanolic *Curcuma longa* extract as a treatment for sand tampan ticks in a rabbit model

**DOI:** 10.14202/vetworld.2020.812-820

**Published:** 2020-04-29

**Authors:** Sobhy Abdel-Shafy, Abdullah D. Alanazi, Hanan S. M. Gabr, Ahmad M. Allam, Hala A. A. Abou-Zeina, Ragab A. Masoud, Doaa E. Soliman, Mohammad Yahya Alshahrani

**Affiliations:** 1 Department of Parasitology and Animal Diseases, Veterinary Research Division, National Research Centre, Dokki, Giza, Egypt; 2 Department of Biological Science, Faculty of Science and Humanities, Shaqra University, P.O. Box 1040, Ad-Dawadimi 11911, Saudi Arabia; 3 Department of Zoology and Agricultural Nematology, Faculty of Agriculture, Cairo University, Giza, Egypt; 4 Department of Tanning Materials and Leather Technology, Chemical Industry Research Division, National Research Centre, Dokki, Giza, Egypt; 5 Department of Entomology, Faculty of Science, Ain Shams University, Cairo, Egypt; 6 Department of Clinical Laboratory Sciences, College of Applied Medical Sciences, King Khalid University, P.O. Box 61413, Abha, 9088, Saudi Arabia

**Keywords:** histopathology, scanning electron microscopy, soft ticks, tick control, turmeric

## Abstract

**Background and Aim:**

The soft tick *Ornithodoros savignyi* is distributed throughout Africa, including Egypt. It primarily attacks camels, cattle, donkeys, and cows; and rarely affects humans. This study evaluated the acaricidal efficacy of ethanolic *Curcuma longa* extract (Turmeric) on the second nymphs of *O. savignyi* and then investigated the safety of this herb in rabbits.

**Materials and Methods:**

The nymphs were immersed in 10, 5, 2.5, 1.25, and 0.625 mg/ml ethanolic *C. longa* extract. An additional group was immersed in ethanol as a control. On the 1^st^, 7^th^, and 15^th^-day post-treatment, the mortality percentages, LC_50_, and LC_95_ were calculated. The ticks exposed to 10mg/ml ethanol *C. longa* extract were investigated by scanning electron microscopy (SEM). Three male New Zealand White rabbits were orally administered 2ml (two doses) of 10mg/ml ethanolic *C. longa* extract, and another three rabbits were orally given two doses of 2ml of absolute ethanol as a negative control. Histopathological examination of the kidney and liver hematology and the kidney and liver function was performed. Chemical analysis of the extract was determined by gas chromatography-mass spectrometry (GC-MS).

**Results:**

The LC_50_ and LC_95_ were 1.31 and 15.07, 1.07 and 8.56, and 0.81 and 6.97mg/ml on the 1^st^, 7^th^, and 15^th^day, respectively. SEM revealed that mamillae and spots on the surfaces of the treated ticks were not discriminating except for some clefts on the surfaces. The histological examination, blood profile, and biochemical analyses revealed no significant differences between the treated and untreated rabbits (p>0.05). GC/MS analysis revealed 50 compounds, and curcumene and tumerone were found to be the major constituents of this ethanolic extract.

**Conclusion:**

The ethanolic *C. longa* extract produced a strong acaricidal effect on the second nymph of *O. savignyi*, and it was safe to use in rabbits.

## Introduction

*Ornithodoros savignyi* is distributed throughout Africa, including Egypt. It primarily attacks animals (camels, cattle, donkeys, and cows); however, rare cases have been found in humans [1,2]. Bites from these ticks cause severe biting stress, paralysis, and toxicosis [1,3]. *O. savignyi* is a vector and a causative agent of many diseases such as *Borrelia*spp.[4,5], Alkhurma hemorrhagic fever virus [6], and Bluetongue virus 8 [7]. Medicinal plants are excellent alternatives to synthetic pesticides as they are eco-friendly, safe for humans and non-target organisms, rapidly biodegradable, and minimally resistant to pests [8,9]. Recent studies have evaluated the effectiveness of crude extracts and essential oils from plants against various tick species [10-12]. Moreover, some commercial acaricides are prepared from plants; and neem and myrrh have exhibited lethal effects against ticks[13,14].

*Curcuma longa* L. is botanically related to the Zingiberaceae family. Turmeric is a spice obtained from the root of *C. longa* that has multiple medicinal properties, including antibacterial, antimalarial, antiviral, anti-aging, anticancer, anti-Alzheimer’s disease, antifungal, antioxidant, and anti-inflammatory, as well as efficacy as a snail repellent[8,15-21]. An ethanolic *C.longa* extract exhibited efficacy against the *Toxoplasma gondii* tachyzoites in mice [22]. Turmeric possesses well-known insecticidal and repellent properties against insect pests. The ethanolic extracts of three *Curcuma* species *Curcuma aeruginosa*, *Curcuma aromatica*, and *Curcuma xanthorrhiza* have displayed mosquito repellent activity [23]. Two compounds isolated from the ethanolic *C. longa* extract ar-turmerone and 8-hydroxyl-ar-turmerone have both exhibited larvicidal effects against the fourth instar larvae of *Culex pipiens* [24].

However, the effect of *C. longa* extract on ticks has not previously been studied. Therefore, this research evaluated the acaricidal efficacy of the ethanolic *C. longa* extract on the soft tick *O. savignyi* and investigated the safety of this herb in rabbits used as an animal model.

## Materials and Methods

### Ethical approval

This study was approved to the ethical standards used in this study and the relevant national and institutional guidelines on the care and use of laboratory animals were approved by the Medical and Veterinary Research Ethics Committee (No, 19014) at the National Research Centre in Egypt.

### Tick collection

**Figure-1:**
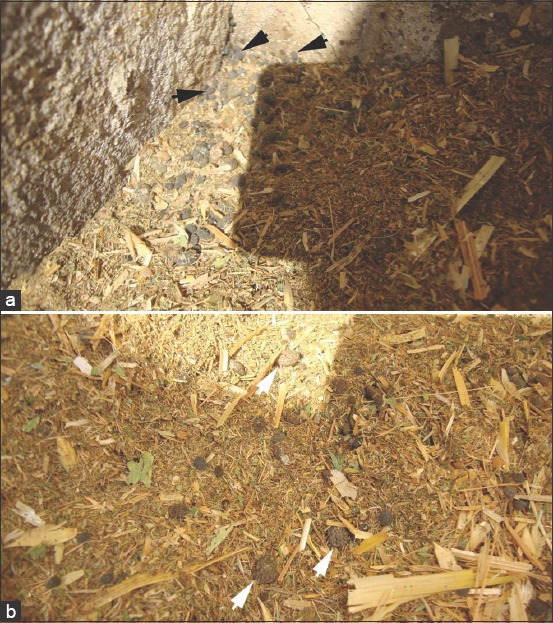
The soft tick *Ornithodoros savignyi* on the ground of the camel yard: (a) Nymphs, (b) adults.

Adult and nymph stage *O. savignyi* ticks were collected from the grounds of the camel market in Shalateen, Egypt (23° 7′ 54″ N, 35° 35′ 8″ E), during September 2018 (Figure-1). Live ticks were taken to the laboratory and identified morphologically using the taxonomic key of Walker *et al*. [1]. The second-instar nymphs were used to evaluate the effects of the ethanolic *C. longa* extract.

### Preparation of the ethanolic *C. longa* extract

Dried rhizomes of *C. longa* were purchased from a local market in Cairo, Egypt. The dried powder was treated with 95% ethanol and evaporated under reduced pressure using a rotatory evaporator. The ethanolic extract was prepared using the dried *C. longa* rhizomes following the method described by Wang and Waller[25]. Briefly, the powder was macerated several times at room temperature for 1week with 95% ethyl alcohol before filtering using Whatman no.1 filter paper. The solvent was removed under reduced pressure at 40°C using a rotary evaporator until a thick paste was formed. This thick paste was considered a concentrated extract. Extracts were stored at 4°C in a refrigerator until use. The different concentrations of the extract were generated by mixing the appropriate volumes of ethanol.

### The toxicity of the ethanolic *C. longa* extract on ticks

Concentrations of the ethanolic *C. longa* extract were prepared at 10, 5, 2.5, 1.25, and 0.625mg/ml. Atotal of 360 *O. savignyi* second nymphs were used during testing; 60 nymphs for each concentration and an additional 60 nymphs as a control group. Each treatment group included three replicates containing 20 nymphs per replicate. The control group contained the same number of ticks, and each was immersed in ethanol as the control treatment. Treatment consisted of immersion of the ticks in the specified extract for 1min before being transferred to the filter paper to absorb the excess extract. Once dry, the nymphs were placed into plastic tubes. These tubes were covered with a piece of cloth secured by a rubber band and incubated at 27°C, 75% relative humidity for 2weeks. The tick mortality was recorded on the 1^st^, 7^th^, and 15^th^days post-treatment. Images of the dead nymphs (from the treatment groups) and live nymphs (control) were acquired using a stereomicroscope. Several dead nymphs from those exposed to the 10mg/ml extract were preserved in 70% ethanol for imaging with a scanning electron microscope (SEM).

### SEM of ticks treated with ethanolic *C. longa* extract

The nymphs that died as a result of the treatment with 10mg/ml ethanol *C. longa* extract were preserved in 10% ethanol on the 1^st^-day post-treatment, and several live nymphs from the control group were preserved in 70% ethanol for comparison. The nymphs were prepared for SEM according to themethod described by Abdel-Shafy [26]. Briefly, the nymphs previously preserved in ethanol were cleaned using a water-glycerol-KCl solution, fixed in glutaraldehyde, immersed in a series of graded alcohol, glued by their dorsal and ventral surfaces to the SEM stub, dried with a dryer (Blazer Union, F1-9496 Blazer/Fürstentun Liechtenstein), mounted on SEM stubs, coated with gold using an S15OA Sputter Coater, and examined by SEM.

### The safety of the ethanolic *C. longa* extract in rabbits

Six male New Zealand White rabbits weighing approximately 2.0kg were used for the *in vivo* evaluation of the ethanolic turmeric extract. Animals were divided randomly into two groups. The test group (n=3) received 2ml of an oral dose of 10mg/ml ethanolic *C. longa* extract. The second group (n=3) was kept as a negative control where the rabbits were orally administered 2ml of absolute ethanol. All rabbits were monitored for 1week and received the treatment twice during this time with a 3-day interval between the two doses. At the end of the experiment, the rabbits were euthanized by carbon dioxide gas asphyxiation with extra confirmation of death by head dislocation, according to the American Veterinary Medical Association guidelines for the euthanasia of animals [27]. The livers and kidneys were retrieved and fixed in 10% neutral buffered formalin for further pathological examination. Paraffin tissue sections at 4-6 μm thickness were prepared and stained with hematoxylin and eosin for histopathological examination. Histological slides were examined by light microscopy [28]. Blood samples were collected from all rabbits at the end of the experiment. Ahemogram test was conducted according to the method described by Weiss and Wardrop [29]. The activities of aspartate aminotransferase and alanine aminotransferase were determined according to the procedure of Reitman and Frankel [30]. Serum urea and creatinine were determined according to the methods of Patton and Crouch [31], and Houot [32], respectively.

### Gas chromatography (GC)/mass spectrometry (MS) analysis

GC (Agilent Technologies 7890A, CA, USA) interfaced with a mass-selective detector (Agilent 7000 Triple Quad, CA, USA) and the Agilent HP-5ms capillary column (30 m×0.25mm ID, 0.25 μm film thickness) were used for analysis. The flow rate was 1mL/min using helium (He) as the carrier. The injector and detector temperatures were 200°C and 250°C, respectively. The acquisition mass range was 50-600. The formulae of the components were identified by comparing the mass spectra and RT information recorded with those of the NIST and WILEY library.

### Statistical analysis

A one-way analysis of variance was used for the statistical analysis between the tick mortality percentages using the F and Tukey’s tests at p<0.05. The statistical analysis between the treated and control rabbits for each blood parameter was performed using a Student’s t-test. All statistical analyses were conducted using SPSS Version 14 (IBM Inc., NY, USA).

## Results

### The toxicity of the ethanolic *C. longa* extract on the *O. savignyi* second nymphs

**Figure-2:**
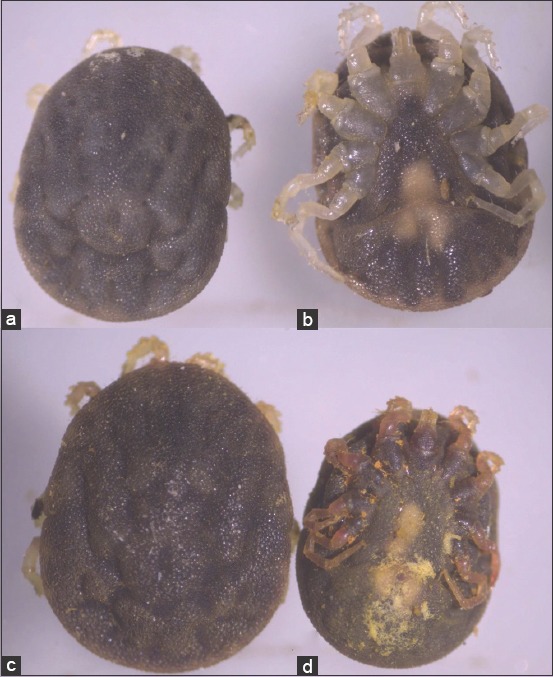
The *Ornithodoros savignyi* second nymphs treated with 10mg/ml ethanolic *Curcuma longa* extract: (a) Dorsal view of nymph exposed to the ethanol only as a control, (b) ventral view of nymph exposed to the ethanol only as a control, (c) dorsal view of nymph immersed in the ethanolic *C. longa* extract, (d) ventral view of nymph immersed in the ethanolic *C. longa* extract.

The primary readout for this experiment included the mortality percentages of the *O. savignyi* nymphs treated with 0.625, 1.25, 2.5, 5, and 10mg/ml ethanolic *C. longa* extract on the 1^st^, 7^th^, and 15^th^days post-treatment (Table-1). Statistical analysis revealed significant differences between the mortalities (p<0.001) recorded for all of the concentrations at each time point post-treatment. The highest mortalities were recorded at concentrations of 2.5-10mg/ml during the 2weeks post-treatment. However, the control group exhibited little mortalities (3.3-6.7%). All treatment concentrations and the control group experienced a non-significant increase in mortality throughout the days of post-treatment (p>0.05). The LC_50_ and LC_95_ slightly decreased over the days of post-treatment 1.31 and 15.07, 1.07 and 8.56, and 0.81 and 6.97mg/ml on the 1^st^, 7^th^, and 15^th^day, respectively (Table-2). There were changes evident in the *O.savignyi* nymphs treated with 10mg/ml of the ethanolic *C.longa* extract compared to that of the controls treated with ethanol. The size and color of the nymphs in the control group remained normal, a dark brown with creamy white legs (Figure-2a and b). In contrast, the treated nymphs were dark red, with swollen bodies, and pink legs (Figure-2c and d).

**Table-1:** Mortality percentages (Mean±SE) of *O. savignyi* second nymphs treated with ethanolic *C. longa* extract.

Concentration (mg/ml)	Day post-treatment		

	1^st^	7^th^	15^th^
10	93.3±6.7^a^	96.7±3.3^ab^	96.7±3.3^a^
5	76.7±8.8^ab^	90.0±0.0^ab^	93.3±3.3^a^
2.5	73.3±12.0^ab^	73.3±12.0^abc^	83.3±12.0^ab^
1.25	50.0±5.8^bc^	56.7±8.8^bc^	63.3±3.3^ab^
0.625	30.0±10.0^cd^	36.7±16.7^cd^	46.7±14.5^b^
Control	3.3±3.3^d^	3.3±3.3^d^	6.7±3.3^c^
Butex	93.3±6.7^a^	100±0.0^a^	-
F value	17.642	16.972	17.650
Probability (p)	<0.001	<0.001	<0.001

Small letters indicate to the significant difference between means using Tukey test at p<0.05. *O. savignyi*=*Ornithodoros savignyi*, *C. longa*=*Curcuma longa*

**Table-2:** LC_50_ and LC_95_ values with their confidence limits for *O. savignyi* second nymphs treated with the ethanolic *C. longa* extract.

Day post treatment	LC_50_ (mg/ml)	LC_95_ (mg/ml)	Confidence limits				Slope±SE

			LC_50_ (mg/ml)		LC_95_ (mg/ml)		
	
			Lower	Upper	Lower	Upper	
1^st^	1.31	15.07	1.05	1.58	10.44	25.61	1.55±0.16
7^th^	1.07	8.56	0.86	1.27	6.42	12.79	1.82±0.17
15^th^	0.81	6.97	0.62	0.99	5.22	10.55	1.76±0.18

*O. savignyi*=*Ornithodoros savignyi, C. longa*=*Curcuma longa*

### SEM analysis of the *O. savignyi* second nymphs

**Figure-3:**
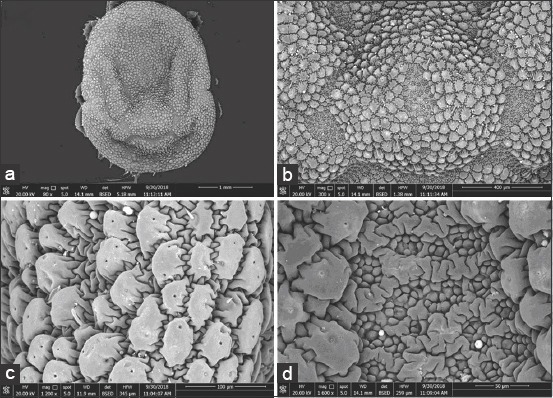
Dorsal view of untreated *Ornithodoros savignyi* second nymph showing: (a) Depressing areas and well distinctive vertical and horizontal grooves, (b) mamillae and spots together, (c) mamilae with one or two holes on their apex, (d) small spots without any holes on their apex.

**Figure-4:**
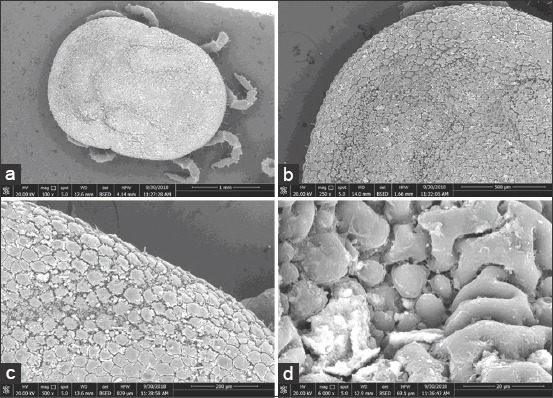
Dorsal view of treated *Ornithodoros savignyi* second nymph by 10mg/ml ethanolic *Curcuma longa* extract showing: (a) Slightly depressing areas and shallow vertical and horizontal grooves, (b) mamillae and spots together, (c) mamilae adhere to each other, (d)undiscriminating spots.

**Figure-5:**
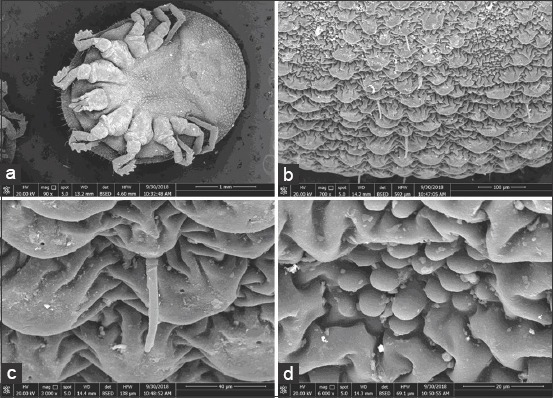
Ventral view of untreated *Ornithodoros savignyi* second nymph showing: (a) Wrinkle integument, mammillae, and spots, (b) mamillae and spots together, (c) mamillae with one hole on their apex, (d) small spots without any holes on their apex.

**Figure-6:**
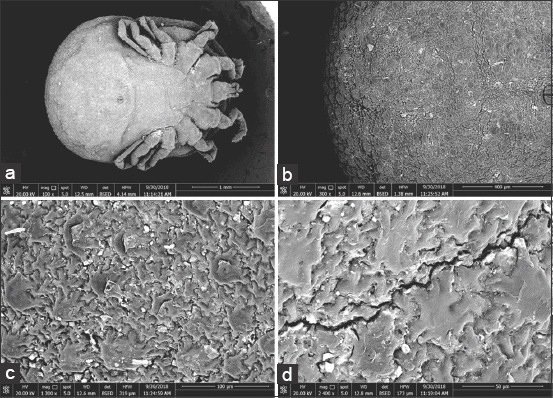
Ventral view of treated *Ornithodoros savignyi* second nymph by 10mg/ml ethanolic *Curcuma longa* extract showing: (a) Indistinctive mammillae and spots, (b) diffused mamillae and spots to form flat surface, (c)mamillae merge each other and spots to form flat surface, (d) long cleft on the surface.

Untreated nymphs exhibited distinctive vertical and horizontal grooves with spots that were visible inside, as well as mammillae all over the dorsal surface (Figure-3a and b). Amammilla is a broad protuberance with one or two holes at the apex (Figure-3c). Aspot is a small tapering protuberance without any holes on the apex (Figure-3d). However, the dorsal surface of the treated nymphs exhibited shallow vertical and horizontal grooves (Figure-4a), and the mammillae appeared to be adhering to each other (Figure-4b and c). Spots on the treated nymphs were an undiscriminating shape (Figure-4d). The ventral surface of the untreated nymphs exhibited wrinkled integument, mammillae, and spots scattered between mammillae throughout the ventral surface (Figure-5a-d), whereas the ventral surface of a treated nymph showed indistinctive mammillae and spots (Figure-6a-d).

### The safety of the ethanolic *C. longa* extract in rabbits

**Figure-7:**
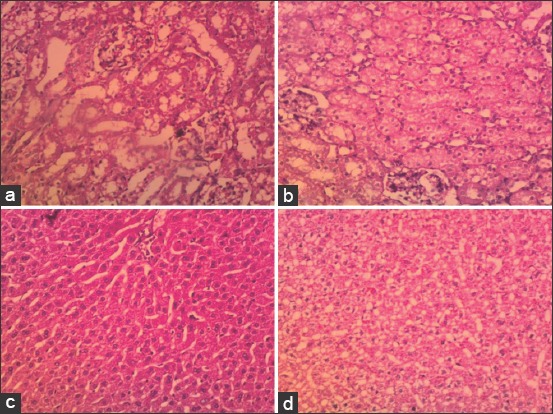
Cross sections in the kidney and liver of the rabbits that orally took 2ml of 10mg/ml ethanolic *Curcuma longa* extract in the treated group and 2ml of absolute ethanol in the negative control group (Hematoxylin and eosin; ×200): (a) Negative control of the kidney showed the normal architecture. (b) The kidney of the rabbit administered *C. longa* extract. (c) Negative control of the liver showed normal architecture with few degenerated areas. (d) The liver of the rabbit administered *C. longa* extract showing moderate reversible liver cell damage in the form of degeneration and congestion.

No major abnormalities were seen in the kidneys of either the negative controls or the treated groups of rabbits (Figure-7a and b). The histological examination of the livers from the negative control rabbits showed no pathological abnormalities (Figure-7c). Liver tissue sections of rabbits that ingested ethanolic *C. longa* extract showed dilation, congestion of the portal, and central veins, areas of vacuolar degeneration, and congestion of the central venules, sinusoids, and portal triad (Figure-7d). The blood profile, kidney function, and liver function were within the normal range in both the control and treated rabbits with non-statistically significant differences (p>0.05) (Table-3).

**Table-3:** Blood profile and liver and kidney functions of rabbits ingested 2 times of 10 mg/ml ethanolic *C. longa* extract.

Parameter	Treated	Control
RBCs	5.52±0.23	5.86±0.01
Hb	12.90±0.17	13.00±0.13
HCT	36.95±0.03	37.95±0.38
MCV	61.55±0.49	61.65±0.95
MCH	21.40±0.46	22.05±0.23
MCHC	34.70±0.40	34.20±0.06
Platelets	452.00±17.90	357.50±12.41
WBCs	6.35±0.20	6.40±0.06
Urea	26.20±0.64	20.15±1.01
Creatinine	1.15±0.03	0.95±0.03
ALT	110.00±9.81	95.50±1.44
AST	58.95±2.34	41.50±2.60

RBCs=Red blood corpuscular counts, Hb=Hemoglobin, HCT=Hematocrit. MCV=Mean corpuscular volume. MCH=Mean corpuscular hemoglobin. MCHC=Mean corpuscular hemoglobin concentration, WBCs=White blood cell counts, ALT=Alanine aminotransferase. AST=Aspartate aminotransferase, *O. savignyi*=*Ornithodoros savignyi, C. longa*=*Curcuma longa*

### Phytochemical analyses of the ethanolic *C. longa* extract

**Figure-8:**
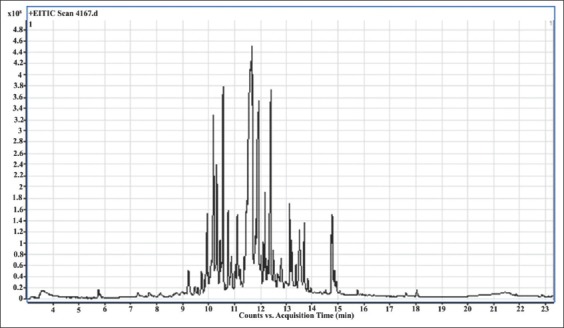
Gas chromatography–mass spectrometry chromatography of the ethanolic *Curcuma longa* extract.

GC–MS analysis of the ethanol extract of *C.longa* identified the major chemical constituents based on the peak retention time (Rt) and percentage area. Atotal of 50 compounds were identified, and the most abundant were as follows (the percentage of abundance is stated in brackets) β-curcumene (30.71), tumerone (7.14), germacone (6.08), β-cedrene (4.59), cedr-8-ene (3.87), β-cubebene (3.34), cis-sesquisabinene hydrate (2.51), p-Cymen-2-ol (2.49), β-himachalene (2.26), Cis-p-mentha-2,8-diene-1-ol (2.1), farnesol (2.1), 7,3`-Dimethyoy-3-hydroxyflavone (2.09), and pinane (2.06) (Table-4 and Figure-8).

**Table-4:** GC-MS of ethanol extract of *C. longa* using library.

No.	Rt (min)	Name of active compounds	% peak area
1	3.5	D-2,3-Butane diol	1.21
2	5.7	o-cymene	0.34
3	6.73	Terpinolene	0.64
4	7.27	α-thujone	0.58
5	7.70	Cis-sabinol	0.33
6	8.13	Ciminaldehyde	0.5
7	8.68	Thymol	0.71
8	8.98	phenol	0.57
9	9.19	Dihydrocurcumene	1.21
10	9.47	Hemellitol	0.38
11	9.57	Cembrene	0.42
12	9.71	α-guaiene	0.96
13	9.82	Longipinene	0.37
14	9.91	β-bisabolene	1.75
15	10.04	γ-gurjunene	0.54
16	10.28	β-Curcumene	1.08
17	10.41	Cuparene	0.47
18	10.45	Cedr-8-ene	3.87
19	10541	β-Cedrene	4.59
20	10.72	Cis-p-mentha-2,8-diene-1-ol	2.1
21	10.81	Cis-p-mentha-6,8-diene-2ol	0.39
23	10.83	Cis-Lanceol	1.05
24	10.87	Geranyl-α-terpinene	0.64
25	11.08	β-Himachalene	2.26
26	11.19	Spathulenol	0.51
27	11.368	Nopol (terpene)	1.08
28	11.65	α-Curcumene	30.71
29	11.78	α-bergamotene	0.55
30	11.81	Zingiberene	0.48
31	11.91	Tumerone	7.14
32	12.00	Longiverbenone	0.66
33	12.102	Trans-Chrysanthenyl acetate	1.18
34	12.155	Caryophyllene oxide	1.77
35	12.257	α-terpinene	0.48
36	12.379	Germacrone	6.08
37	12.493	Elemol	0.93
38	12.66	Geranyllinalool	0.86
39	12.786	Terpinylformate	1.51
40	12.969	Cis-sesquisabinene hydrate	2.51
41	13.108	Corymbolone	1.51
42	13.365	α-lonone	0.59
43	13.495	p-Cymen-2-ol	2.49
44	13.678	7,3`-Dimethyoy-3-hydroxyflavone	2.09
45	14.738	Pinane	2.05
46	14.774	Farnesol	2.1
47	14.86	5β, 7βH, 10 α-eudesm-11-en-1a-ol	0.89
48	15.74	Phytol	0.38
49	21.456	Stigmasterol	0.46
50	22.85	β-sitosterol	0.22

*C. longa*=*Curcuma longa,* GC-MS=Gas chromatography–mass spectrometry

## Discussion

Alcohols are solvents that have demonstrated superior extractive power for almost all-natural substances of low molecular weight, such as alkaloids, saponins, and flavonoids [33]. The ethanolic extracts from the rhizome of *C. longa* L. possessed a wide variety of biological activities related to the treatment and prevention of various clinical conditions [34]. The *C. longa* rhizome extract was reportedly a more potent antimicrobial agent than the leaf extract [18], and higher concentrations of curcumin and antioxidant activity were obtained when ethanol was used as the solvent for extraction [17]. Therefore, this study evaluated the effects of the crude ethanolic extract of *C. longa* L. against the second nymphs of the soft tick *O. savignyi*, which was found in large numbers on the grounds of a local camel yard in Egypt. The safety of this extract in rabbits was also evaluated. The *C.longa* L. rhizome was selected for extraction and evaluation against this tick species for three reasons: (1) It is available in the local market in a powder form for a low price, (2) the previous studies found that different *C. longa* L. derived materials exhibited strong insecticidal activity on various insect pests such mosquitoes, and (3) no previous studies had evaluated the effects of *C. longa* L. extract on this tick species.

In this study, it was found that the *O. savignyi* second nymphs were highly susceptible to the ethanolic *C. longa* extract as 2.5-10mg/ml of the extract caused 73.3-96.7% mortality in the nymphs after 1week of treatment. There were non-statistically significant differences (p>0.05) between the mortality range recorded with the extract and the mortality range of the reference acaricide (93.3 and 100%). The calculated LC_50_ values of the extract used in this research were 1.31, 1.07, and 0.81mg/ml on the 1^st^, 7^th^, and 15^th^days, respectively. This indicated that the toxicity of the ethanolic *C. longa* extract increased over time. Another study[35] had evaluated the effects of *C. longa* on ticks, using essential oils rather than extract, and their results were similar to the results of the current study[35]. These researchers found that of 11 Brazilian essential oils, *C. longa* essential oil produced the best results against *Rhipicephalus* (*Boophilus*) *microplus* ticks, with LC_50_ values of 10.24 and 0.54mg/mL against the females and larvae, respectively[35]. Furthermore, the essential oils of *Curcuma* spp. demonstrated highly larvicidal activity against *Aedes aegypti* and *Anopheles gambiae* with LC_50_ values of 36.30 and 149ppm, respectively [36,37]. In addition, petroleum ether extract from *C. aromatica* showed larvicidal activity against *Culex quinquefasciatus* at an LC_50_ value of 11.41 [38]. Moreover, two compounds, ar-turmerone and 8-hydroxyl-ar-turmerone isolated from the ethanolic extracts of the *C. longa* root, exhibited larvicidal activities against the fourth-instar larvae of *C. pipiens pallens* following 24h of treatment; LC_50_ values were 138.86 and 257.68ppm, respectively [24].

Changes to the morphological features of the ticks were observed on treatment with the ethanolic *C. longa* extract, cuticles became darker, and the ticks’ legs changed from a creamy white color to a pinkish hue. This suggested that the extract could penetrate the tick’s cuticle, causing damage to the guts, which spread throughout the tick’s body, including the legs. Furthermore, the SEM images revealed additional changes that occurred on the surfaces of the ticks that were exposed to the ethanolic *C. longa* extract. The mammillae of the treated ticks appeared to overlap, the spots on the surface were different, and there were many crevices on the surfaces of treated ticks. These changes might be due to swelling of the tick’s body. The ethanolic *C. longa* extract might cause inhibition of the acetylcholinesterase activity as it contained high levels of polyphenols [39]. Consistent with the effects seen in the present study, Chaaban *et al*. [40] observed that *C. longa* essential oil caused damage to the tick cuticles, midgut, and brain, and produced a fattening of the body of the third-instar *Lucilia cuprina* (Diptera: Calliphoridae) larvae.

Two doses of 2ml (10mg/ml ethanolic) *C.longa* extract were administered to rabbits with 3days between doses to assess the safety of the extract in a mammalian system. Biochemical blood parameters were measured for assessment. These parameters were related to health status and considered good indicators of the physiological, pathological, and nutritional status of the animals [41]. The results confirmed the safety of the extract in rabbits. There were no significant changes observed in the kidney function tests or the histological analysis in rabbits that received the extract compared to the control group. Furthermore, there were no significant changes in the blood profile or liver functions between the treated and control rabbits. The results agreed with the findings in the literature [42], in which the authors found no adverse effects on the erythrocytes, leukocytes, or the concentrations of blood constituents. However, slight changes were observed in the livers of the *C. longa* extract-treated rabbits compared with the control group. Mesa *et al*.[43] found that oral administration of the *C.longa* extract reduced susceptibility to the oxidation of erythrocytes and liver microsome membranes. Turmeric’s hepatoprotective effect was primarily a result of the antioxidant properties and the ability to reduce the formation of the pro-inflammatory cytokines. Moreover, curcumin administration significantly decreased liver injury [43].

In the present study, the GC–MS analyses of the ethanolic *C. longa* extract revealed 50 compounds, of which the 13 most abundant comprised the majority of the sample. Many of these compounds were major components of essential oils. The natural phytochemicals of *C. longa* have been associated with anticancer, anti-inflammatory, neuroprotective, anti-Alzheimer’s, and antioxidant activities. Furthermore, the volatile oil of turmeric has been widely used in cosmetics or health products and possesses antimicrobial, antifungal, and anti-arthritic properties. The natural phytochemicals of *C. longa* are also considered to affect a variety of biological activities [44,45]. The two most abundant compounds of the ethanolic *C. longa* extract were curcumene and tumerone, which might be responsible for the acaricidal activity against the second nymphs of *O. savignyi* in the present study. Furthermore, some studies have identified that other compounds isolated from the ethanolic extracts of *C. longa* root have exhibited insecticidal activity against some insects such as mosquitoes. Liu *etal*.[24] isolated ar-turmerone and 8-hydroxyl-ar-turmerone from the *C. longa* root ethanolic extracts and identified larvicidal activities against the fourth-instar larvae of *C. pipiens*. Furthermore, Ali *et al*. [46] discovered that the ethanolic extract of *C.longa* was comprised 15.4% of ar-turmerone, 6.6% of bisdesmethoxycurcumin, 6.1% of desmethoxycurcumin, and 22.6% of curcumin.

## Conclusion

The ethanolic curcumene and tumerone were the most abundant constituents identified in the extract. The ethanolic *C. longa* extract elicited a strong acaricidal effect on the second nymphs of the soft tick *O. savignyi*, while no adverse effects were found in the rabbits. Therefore, this extract could be used in pest management applications against ticks found on animals.

## Authors’ Contributions

All authors shared in the study plan. SA, HSMG performed the bioassay of the ethanolic *C. longa* extract on the ticks. SA, HSMG, and AMA participated in the administration of the ethanolic *C. longa* extract in the rabbits. SA and DES photograph the ticks by light microscope and scanning electron microscope and analyzed the data. AMA analyzed the blood and performed the cross-sections of the rabbit organs. HAAA and RAM participated in the chemical analyses of the ethanolic *C. longa* extract. SA, AMA, HAAA, RAM, ADA, and MYA shared in writing the manuscript. All authors revised and approved the final manuscript.
